# PCR reveals significantly higher rates of *Trypanosoma cruzi *infection than microscopy in the Chagas vector, *Triatoma infestans*: High rates found in Chuquisaca, Bolivia

**DOI:** 10.1186/1471-2334-7-66

**Published:** 2007-06-27

**Authors:** Juan Carlos Pizarro, David E Lucero, Lori Stevens

**Affiliations:** 1 Department of Biology, University of Vermont, 109 Carrigan Drive, Burlington, VT 04505, USA; 2 Facultad de Bioquímica, Universidad de San Francisco Xavier de Chuquisaca, Sucre, Bolivia

## Abstract

**Background:**

The Andean valleys of Bolivia are the only reported location of sylvatic *Triatoma infestans*, the main vector of Chagas disease in this country, and the high human prevalence of *Trypanosoma cruzi *infection in this region is hypothesized to result from the ability of vectors to persist in domestic, peri-domestic, and sylvatic environments. Determination of the rate of *Trypanosoma *infection in its triatomine vectors is an important element in programs directed at reducing human infections. Traditionally, *T. cruzi *has been detected in insect vectors by direct microscopic examination of extruded feces, or dissection and analysis of the entire bug. Although this technique has proven to be useful, several drawbacks related to its sensitivity especially in the case of small instars and applicability to large numbers of insects and dead specimens have motivated researchers to look for a molecular assay based on the polymerase chain reaction (PCR) as an alternative for parasitic detection of *T. cruzi *infection in vectors. In the work presented here, we have compared a PCR assay and direct microscopic observation for diagnosis of *T. cruzi *infection in *T. infestans *collected in the field from five localities and four habitats in Chuquisaca, Bolivia. The efficacy of the methods was compared across nymphal stages, localities and habitats.

**Methods:**

We examined 152 nymph and adult *T. infestans *collected from rural areas in the department of Chuquisaca, Bolivia. For microscopic observation, a few drops of rectal content obtained by abdominal extrusion were diluted with saline solution and compressed between a slide and a cover slip. The presence of motile parasites in 50 microscopic fields was registered using 400× magnification. For the molecular analysis, dissection of the posterior part of the abdomen of each insect followed by DNA extraction and PCR amplification was performed using the TCZ1 (5' – CGA GCT CTT GCC CAC ACG GGT GCT – 3') and TCZ2 (5' – CCT CCA AGC AGC GGA TAG TTC AGG – 3') primers. Amplicons were chromatographed on a 2% agarose gel with a 100 bp size standard, stained with ethidium bromide and viewed with UV fluorescence.

For both the microscopy and PCR assays, we calculated sensitivity (number of positives by a method divided by the number of positives by either method) and discrepancy (one method was negative and the other was positive) at the locality, life stage and habitat level. The degree of agreement between PCR and microscopy was determined by calculating Kappa (*k*) values with 95% confidence intervals.

**Results:**

We observed a high prevalence of *T. cruzi *infection in *T. infestans *(81.16% by PCR and 56.52% by microscopy) and discovered that PCR is significantly more sensitive than microscopic observation. The overall degree of agreement between the two methods was moderate (Kappa = 0.43 ± 0.07). The level of infection is significantly different among communities; however, prevalence was similar among habitats and life stages.

**Conclusion:**

PCR was significantly more sensitive than microscopy in all habitats, developmental stages and localities in Chuquisaca, Bolivia. Overall we observed a high prevalence of *T. cruzi *infection in *T. infestans *in this area of Bolivia; however, microscopy underestimated infection at all levels examined.

## Background

Chagas disease, caused by the parasite *Trypanosoma cruzi*, was ranked in the 1990's as the most serious parasitic disease in Central and South America in terms of social and economic impact [1]. In the early 1990s more than 16 million people were infected [2]. Recent progress under the aegis of the Southern Cone Initiative has been highly effective in Uruguay, Chile, most of Brazil, four provinces in Argentina, and one department in Paraguay [3,4] but in other high-risk areas success has been more modest. Active *T. cruzi *transmission is still present in Bolivia especially in the Departments of Cochabamba and Chuquisaca, located in the Andes. These areas report the highest prevalence in Bolivia for children less than five years of age, 22% and 35% respectively [5]. In South America, *T. infestans *is the most widespread vector of *T. cruzi*. The Andean valleys of Bolivia are the only reported location of sylvatic *T. infestans *[6,7], and the high human prevalence in this region is hypothesized to result from the ability of vectors to persist in domestic, peri-domestic, and sylvatic environments.

Determination of the rate of *Trypanosoma *infection in its triatomine vectors is an important element in programs directed at reducing human infections [8,9]. Traditionally, *T. cruzi *has been detected in insect vectors by direct microscopic examination of intestinal contents or dissection and analysis of the entire bug [10-12]. These conventional microscopy assays offer fairly reliable diagnosis with relatively low cost, however the method is best performed in living insects since once the insect is dead the parasite is nearly impossible to detect in insect feces [13]. In many endemic countries, community-based insect collections monitor house infestation/re-infestation and subsequent determination of the rate of insect infection with *T. cruzi*. These programs include the collection of insects in geographically very isolated communities with seasonal road infrastructure for transportation of specimens to facilities with equipment for microscopic examination. Vectors, often stored for months after collection [14], frequently die before microscopic analysis is feasible. Moreover, microscopic observation is a laborious process, requiring about 10 minutes for extrusion of the abdomen to obtain the sample, mounting the plate and scanning per insect, so that a diligent and assiduous worker can assay about 50 specimens per day whilst incurring eyestrain and back pain. Repeatability is likely to wane through the day and is known to vary among observers. In addition, larger late instars and adult specimens are much easier to exude feces from for analysis; smaller instars are often ignored because of handling difficulties. Therefore, a fast and sensitive parasitological assay capable of working on degraded samples would enhance our ability to monitor the presence of *T. cruzi *in the triatomine vector of Chagas disease.

It is well known that PCR-based detection from feces or urine of reduviid bugs, and from blood samples of mammalian hosts is one of the most cost and time effective techniques and is usually superior in accuracy. Molecular assays based on the polymerase chain reaction (PCR) have been proposed as an alternative for parasitic detection of *T. cruzi *infection in vectors [10]. Several primers complementary to the conserved region of the kinetoplast-minicircle part of the mitochondrial DNA have been used in PCR based assays in order to detect this parasite in vertebrate blood samples [8,11,15] and in fecal content of triatomines [16-18]. However, it has been suggested that the technique using the TCZ1 and TCZ2 primers that amplify a 188 bp of a 195-bp repetitive nuclear sequence is the most sensitive compared with methods using S35 and S36 primers to amplify a 330-bp minicircle sequence [10,19]. There are approximately 1.8× copies of the 195-bp element per organism relative to the amplifiable minicircle regions making this assay a highly sensitive method for detecting small numbers of parasites [10].

Extensive spraying with insecticide has reduced *T. infestans *populations and consequently its *T. cruzi *infection [20], which is known to vary among hosts [21]. Scarce information is available on infection levels of *T. infestans *collected in the field taking into account early nymphal stages and considering vector habitat. We have compared a PCR assay, based on a repetitive 195-bp segment of nuclear DNA, to direct microscopic observation for diagnosis of *T. cruzi *infection. We examined 152 specimens including all nymphal instars and both sexes of adult *T. infestans*. Live insects were collected in and around houses, and in chicken coops as part of the community-based anti-Chagas programme from five communities in Chuquisaca, Bolivia (Table 1).

**Table 1:** Geographic origin, habitat and developmental stage of *T. infestans *from Chuquisaca, Bolivia, included in the study

		Stage							
		
Locality	Habitat	N1	N2	N3	N4	N5	AF	AM	Total number
Jackota	Goat corral		5	4	4	12	3	6	34
Zurima	Domicile	6	10	3		4	8	11	42
	Peri-domicile	2	8	9	1	6	7	5	38
	Chicken-coop	2	1	2		1			6
Serrano	Domicile		1	4	1	3		1	10
	Peri-domicile			1					1
	Chicken-coop			1	1	1			3
Capilla Llave	Peri-domicile		1	5	1	7			14
Carbajal	Chicken-coop						2	2	4
Total number		10	26	29	8	34	20	25	152

A = Adult; F = Female; M = Male; N1–N5 = First to fifth nymphal instars

## Methods

### Insect collection

Insects were collected from five rural communities in the Bolivian highlands (2200–2600 meters above sea level), Department of Chuquisaca, Bolivia: Capilla Llave (18°58'46"S; 64°42'21"W), Jackota (19°04'00"S; 64°48'00"W), Zurima (18°45'00"S; 65°04'60"W), Serrano (19°06'00"S; 64°22'00"W) and Carbajal (19°21'40"S; 65°18'14"W). The topography includes numerous valleys and small plateaus with very diverse climates [22]. Plateaus receive less than 500 mm precipitation annually and their average temperature is less than 10°C. The valleys present more moderate climates, with average temperature of 18°C and 500 – 600 mm precipitation. The region has a history of high rates of human infection for *T. cruzi *and high rates of house infestation by *T. infestans *[23]. Most houses have one or two adjacent bedrooms and a kitchen. The peri-domestic area comprises several structures neighboring the house, including pigpens; goat, sheep and cow corrals that we included in the category of "corrals" in our study; and chicken coops. The construction is generally of adobe walls for houses and fences of corrals, combined with roofs of earth covered with split cane or ceramic tile. Thorn scrub branches are often also used in corrals atop adobe blocks to limit livestock movement and protect adobe from rain.

Nymph and adult insects were collected from inside homes, as well as in their immediate vicinity, by the residents under the community-based anti-Chagas control program. Collection date, locality, and habitat (domestic, peri-domestic, chicken coop) were recorded and live insects, in plastic cups with folded paper, were transported to the vector control laboratory at the Servicio Departamental de Salud (SEDES) de Chuquisaca for microscopic analysis. After analysis, insects were placed in containers with 96% ethanol and shipped to the University of Vermont, USA for species and nymphal instar or adult sex identification [24] and DNA analysis.

### Microscopy

Rectal contents were removed through abdominal extrusion and microscopically examined for the presence of *T. cruzi *[25]. For microscopic observation, a few drops of the rectal content were diluted with saline solution and then compressed between a slide and a cover slip. Fifty microscope fields were examined for the presence of motile parasites at 400× magnification.

### DNA extraction

A new razor blade was used to collect 25 mg of tissue from the posterior part of each insect's abdomen. The cut was made as close to the posterior as possible to avoid the stomach, which has been shown to inhibit the PCR reaction [13]. The tissue was placed in a 1.5 ml micro centrifuge tube using forceps that were placed in bleach and rinsed in water after each extraction to minimize contamination. Subsequent DNA extraction used the DNeasy kit (Qiagen, Valencia CA) following the protocol for animal tissues with 24-hour lysis. DNA concentration was measured using a Nanodrop 1000 spectrophotometer (Nanodrop, Bethesda, MD).

### PCR amplification

**Figure 1:**
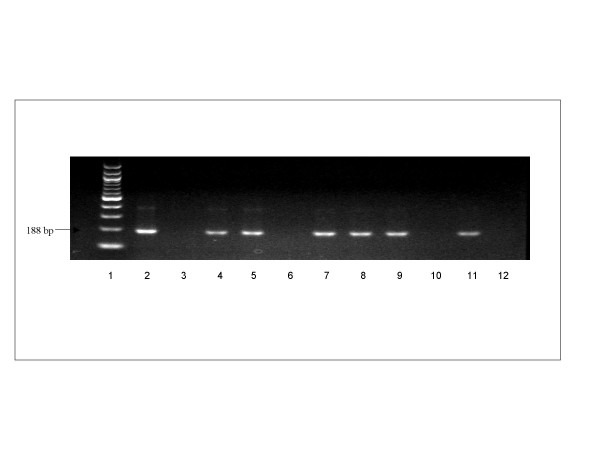
The 188-bp PCR product amplified from *T. cruzi *DNA in 2% agarose gel stained with ethidium-bromide. Lane 1, 100 bp ladder; lane 2, positive control; lane 3, negative control, water as template; lanes 6, 10 and 12, negative results; lanes 4, 5, 7, 8, 9 and 11, positive results.

The primers used for the PCR amplification, were designated as TCZ1 (5' – CGA GCT CTT GCC CAC ACG GGT GCT – 3') and TCZ2 (5' – CCT CCA AGC AGC GGA TAG TTC AGG – 3') [10]. Reactions contained 10 μM of each primer, one Ready-To-Go PCR bead (Amersham Bioscience, Piscataway, NJ) and molecular biology grade water to 25 μl. We added 50–100 ng DNA to each reaction; however because DNA extracted from vectors could contain multiple types of DNA (e.g., insect + *T. cruzi*) we could not quantify the amount of *T. cruzi *DNA in each reaction. The PCR protocol included an initial denaturation at 94°C for 10 m; 30 cycles of 94°C for 20 s (to denature the template), cooling to 57°C for 10 s (to anneal the primers), and heating to 72°C for 30 s (for DNA synthesis) and final extension for 7 m at 72°C. Amplicons were chromatographed on a 2% agarose gel with a 100 bp size standard, stained with ethidium bromide and visualized using UV fluorescence. Positive controls confirmed the ability of primers to amplify *T. cruzi *target DNA and distilled water was used as template for negative controls (Figure 1). To rule out PCR inhibition by some components of the blood meal, some PCR-negative samples were spiked with 0.1 ng of *T. cruzi *DNA obtained from a pool of positive samples previously tested by sequencing.

## Results

Overall we observed a high prevalence of *T. cruzi *infection in *T. infestans *(81.16% by PCR and 56.52% by microscopy). The sensitivity was examined using the χ^2 ^test [26]. The sensitivity (number of positives by a method divided by the number of positives by either method) of PCR, 100%, is significantly higher than microscopy, 69.64%, (χ^2 ^= 44.45. P < 0.0001, Table 2) [26].

**Table 2:** Comparison of PCR and Microscopy for *T. cruzi *detection in *T. infestans *by developmental stage* in Chuquisaca, Bolivia

Stage	PCR %	Microscopy %	Sample size	P-value**	Discrepancy %	Kappa	s.e.
N1	80.00	60.00	10	0.05	20.00	0.54	0.26
N2	80.00	48.00	25	0.02	32.00	0.37	0.14
N3	82.61	47.83	23	0.03	34.78	0.32	0.14
N4	100.00	50.00	6	0.39	50.00	NA	NA
N5	80.00	70.00	30	<0.0001	10.00	0.74	0.14
A	79.55	56.82	40	0.0002	24.44	0.38	0.13
Total	81.16	56.52	138	<0.0001	24.64	0.43	0.07
Sensitivity	100.00	69.64		<0.0001			

* Serrano and Carbajal not included in the analysis

N1–N5 = First to fifth nymphal stages; A = Adults; PCR = Polymerase Chain Reaction;

Discrepancy = PCR positive and Microscopy negative; NA = Non applicable

** P-value = Probability that the differences in infection rates between techniques is due to chance

The degree of agreement was moderately high at 75.36%. In 24.64% of the cases where microscopy was negative the PCR was positive. Whereas discrepancy (PCR negative microscopy positive) was negative. The degree of agreement (1 – discrepancy) between PCR and microscopy was estimated by the agreement statistic, Kappa, *κ*:

κ=po−pe(1−pe).
 MathType@MTEF@5@5@+=feaafiart1ev1aaatCvAUfKttLearuWrP9MDH5MBPbIqV92AaeXatLxBI9gBaebbnrfifHhDYfgasaacH8akY=wiFfYdH8Gipec8Eeeu0xXdbba9frFj0=OqFfea0dXdd9vqai=hGuQ8kuc9pgc9s8qqaq=dirpe0xb9q8qiLsFr0=vr0=vr0dc8meaabaqaciaacaGaaeqabaqabeGadaaakeaaiiGacqWF6oWAcqGH9aqpdaWcaaqaaiabdchaWnaaBaaaleaacqWGVbWBaeqaaOGaeyOeI0IaemiCaa3aaSbaaSqaaiabdwgaLbqabaaakeaadaqadaqaaiabigdaXiabgkHiTiabdchaWnaaBaaaleaacqWGLbqzaeqaaaGccaGLOaGaayzkaaaaaiabc6caUaaa@3D9C@

**Figure 2:**
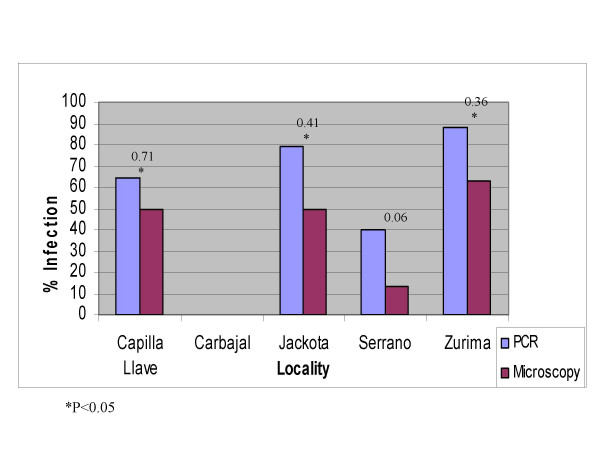
Agreement between PCR and microscopy by locality; numbers above bars are values of Kappa.

**Figure 3:**
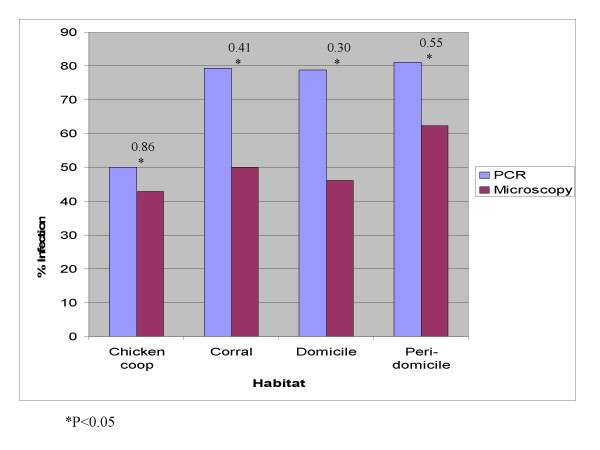
Agreement between PCR and microscopy by habitat; numbers above bars are values of Kappa.

Where *p*_*e *_is the proportion expected to agree by chance and *p*_*o *_is the overall agreement [27]. Low *κ *values represent slight agreement, and higher values indicate substantial agreement. If the two methods give conflicting results *κ *can be negative. Overall, the two methods have moderate agreement *κ *= 0.43, s.e. = 0.07 (Table 2) [26]. Both techniques significantly differ in all communities except in Serrano, even though in this community the Kappa value was small (Figure 2). The difference between PCR and microscopy was significant in all habitats (Figure 3).

**Figure 4:**
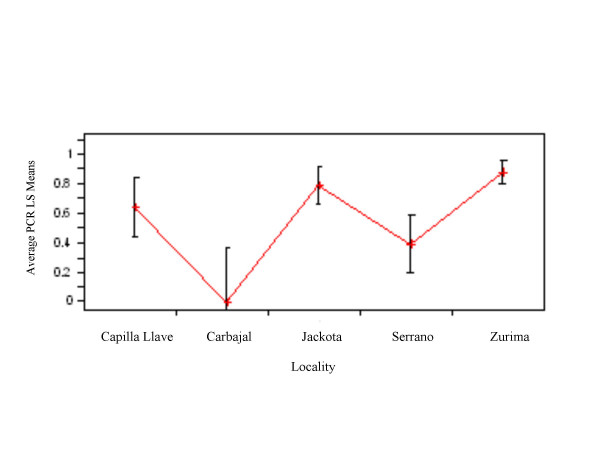
Comparison of PCR results by locality; numbers on the Y-axis are adjusted means for PCR results.

The sample sizes by locality, habitat and life stage are shown in Table 1. Because of the unequal sampling we tested the prevalence of infection for effects of age, habitat and locality separately. In three cases there was a reasonable sample size for specimens from a single locality and habitat; therefore, we tested for an age affect using these groups. Serrano had only one adult and Carbajal had only adults, thus we excluded these two localities from the age effect analysis. The value for the logistic regression [26] of PCR result vs age was not significant for each of these groups; Jackota goat corral, Zurima domicile and Zurima peri-domicile (χ^2 ^= 0.74, P > 0.05; χ^2 ^= 3.09, P > 0.05; χ^2 ^= 0.33, P > 0.05), demonstrating that likelihood of infection does not vary with age. Because the age-effect was not significant, we pooled the age groups to examine the effects of habitat. Vectors from the three habitats in Zurima, domestic, peri-domestic and chicken coop, did not differ in prevalence of infection (Likelihood ratio χ^2 ^= 1.80, P > 0.05). Variation among the localities, tested after pooling across the non-significant habitat and life stage factors, showed that two communities Serrano and Carbajal had significantly lower prevalence than the others (F = 10.09; d.f. = 4; P < 0.0001, Figure 4). The efficacy of PCR vs microscopy may be affected by age, habitat or localities; for example, microscopic detection may be more accurate in larger life stages. We computed the accuracy of microscopy vs nymphal life stage for data pooled across habitats for the three communities with similar prevalence (Zurima, Jackota and Capilla Llave, data not shown). There was no significant association between age and accuracy (χ^2 ^= 2.00, P > 0.05).

## Discussion

We have compared microscopy and PCR for the main vector of Chagas disease in Bolivia taking into account four different habitats and the life stage of the vectors examined from five localities in Northern Chuquisaca. The major finding is that the PCR is significantly more sensitive than microscopy and this result is not dependent on the life stage (or size) or habitat of the insect examined. Except for Serrano, all other communities showed that PCR was significantly more sensitive than microscopy.

The high level of infection of insects collected in the field is similar to a study done on *T. infestans *in the Paraguayan endemic zone for Chagas disease. In Paraguay, slightly more than 84% of the samples were positive by molecular analysis using PCR-Southern hybridization [14], compared to 81% in the present study. However the discrepancy varied between the two studies, in Paraguay only 26% were positive by direct microscopic observation whereas our study detected more than 56% as infected by microscopy.

Not all studies have shown PCR to be more sensitive. In a study published in 1995 on field collected *T. infestans *from Bolivia, the discrepancy between the two methods was 14.70%: microscopy was significantly more sensitive than PCR when amplifying the hypervariable region of kinetoplast DNA minicircles of *T. cruzi *[17]. Usually PCR is the more sensitive method, for example a 1996 report of *T. cruzi *detection in *T. infestans *fed on patients with chronic Chagas disease showed a discrepancy of 46% with respect to the microscopic detection [8] and in 1999 is was reported that in *Rhodnius prolixus *from Guatemala, using primers for the conserved region of the kinetoplast minicircles, the discrepancy was 34.9% [13]. The relative sensitivity of PCR is even larger when analyzing dead insects. In *T. dimidiata*, higher rates of infection were revealed when studying rectal samples as compared to those from intestine and stomach; although the difference was not significant, prevalence detected by PCR was higher than for microscopy (Discrepancy = 14.5%) [13]. The use of different primers and sample processing procedures [16], improvements in PCR detection limits or differences in the microscopes, and variation in intensity of *T. cruzi *infection in *T. infestans *[13] could all contribute to differences in sensitivity of the two methods. Considering the results of this and other studies, the most sensitive method appears to be using TCZ1 and TCZ2 primers directed to amplify a 188 bp of the 195-bp repetitive nuclear sequence and sampling the rectum of the bug.

The prevalence of infection showed to be significantly different among the communities considered. However, there were not enough insects collected per locality to adequately compare prevalence of infection among different communities except for Jackota and Zurima. Interestingly there were no differences among the different habitats and the accuracy did not vary with vector life stage. However, the discrepancy between both techniques was significant by habitat and locality, except in Serrano. We found high prevalence of infection in younger nymphs. This could be a reflection of the early exposure to infection in areas with high prevalence of *T. cruzi *infection in humans and other hosts, which is the case of this area of Bolivia. This fact provides additional evidence of the importance of younger nymphs in parasite transmission [9].

A potential problem with PCR detection of *T. cruzi *is the occurrence of false-positive results caused by contamination of reaction mixtures with amplicon from previous reactions that used the same set of primers [19]. Because of the high percentage of specimens with negative microscopy and positive PCR, we were careful to include controls consisting of distilled water to test for the possibility of spurious results caused by contamination. We are confident that this problem is absent or minimal in our study because of the consistent lack of amplification in all negative controls. Water, rather than DNA extracted from uninfected insects, for the negative control has the advantage that it lacks PCR inhibitors that may be present in other substrates, ruling out contamination in a reaction mixture most likely to amplify DNA. To further reduce the chances of cross-contamination, only one sample per insect was obtained and the dissection and PCR processing were performed in separate areas of the laboratory. The fragment size of positive samples and positive control reactions were as expected for *T. cruzi *(Figure 1). In addition, PCR negative samples spiked with 0.1 ng of *T. cruzi *DNA showed amplification after a second PCR. Comparison of the sequence of our PCR products to those in GenBank confirmed that the amplified product was *T. cruzi *DNA.

The usual standard for microscopic examination for parasite prevalence is based in the observation of 50 fields. For *T. cruzi *infection in *T. infestans *this takes approximately 10 minutes per sample. It is important to note that samples must be fresh because observers are looking for motile parasites. Difficulty in maintaining live insects or in maintaining live parasites within insects may lead to an underestimation. Observer fatigue may occur when many samples are processed in a single day, resulting in failure to notice parasites in some samples. This may be especially true when infection intensity is low as is the case in insects fed on chickens and other peri-domestic hosts. Low prevalence is often associated with low infection intensity [21]. In our study this may explain in part the disagreement between PCR and microscopy by locality as the difference in *T. cruzi *prevalence was significant. Another issue with microscopic observation is that it is usually difficult to obtain a fecal drop from first and second instar nymphs leading to an undersampling in estimation of the rate of infection in these stages, or observers may bias sampling towards later instars. These are not issues in the PCR assay.

We have shown that PCR is a useful method to detect *T. cruzi *infection in the insect vector *T. infestans*. The procedure works well even from samples taken from dead insects, in which case microscopy has been shown to be inadequate [13]. The PCR can also be used to monitor the infection status of triatomines infesting rural dwellings by examining only the feces left on paper sensors hung on the walls of the houses [9]. The abundance and specificity of the 195-bp repetitive element make it an ideal target sequence for primer-directed amplification of *T. cruzi *DNA. Another advantage of PCR over microscopy is that the sample can be processed long after the insect is collected or the sample taken from the abdomen. This is particularly useful in endemic countries, where the collection of insects is often in remote areas and specimens are transported for analysis days to weeks later. The DNA extractions, amplifications, and analyses of dozens of insects can be conducted simultaneously and the numbers of parasites in individual insects could be estimated by quantitative PCR [10].

## Conclusion

The central finding of the present study is that the PCR is significantly more sensitive than microscopy in all habitats, developmental stages and localities in Chuquisaca, Bolivia. Overall we observed a high prevalence of *T. cruzi *infection in *T. infestans *in this area of Bolivia; however, microscopy underestimated infection at all levels examined. This study provides additional evidence of the importance of the PCR assay for the determination of the rate of *Trypanosoma *infection in its triatomine vectors.

## Competing interests

The author(s) declare that they have no competing interests.

## Authors' contributions

JCP: participated in the conception and design of the study, carried out the microscopic observation, insect classification, DNA extraction, PCR analysis, data analysis and drafted the manuscript. LS: participated in the conception and design of the project, optimization and DNA extraction for the PCR analysis, data analysis and preparation of the manuscript. DL: carried out the PCR-based detection assay. All authors read and approved the final manuscript.

## Pre-publication history

The pre-publication history for this paper can be accessed here:


